# Therapeutic MSC exosomes are derived from lipid raft microdomains in the plasma membrane

**DOI:** 10.3402/jev.v2i0.22614

**Published:** 2013-12-23

**Authors:** Soon Sim Tan, Yijun Yin, Tricia Lee, Ruenn Chai Lai, Ronne Wee Yeh Yeo, Bin Zhang, Andre Choo, Sai Kiang Lim

**Affiliations:** 1 Institute of Medical Biology, A*STAR, Singapore; 2 Bioprocessing Technology Institute, A*STAR, Singapore; 3 Department of Surgery, YLL School of Medicine, National University of Singapore, Singapore

**Keywords:** exosomes, transferrin, cholera toxin B, lipid rafts, clathrin mediated endocytosis, mesenchymal stem cells

## Abstract

**Background:**

Mesenchymal stem cell (MSC) was previously shown to secrete lipid vesicles that when purified by high performance liquid chromatography as a population of homogenously sized particles with a hydrodynamic radius of 55–65 nm reduce infarct size in a mouse model of myocardial ischemia/reperfusion injury. As these vesicles exhibit many biophysical and biochemical properties of exosomes, they were identified as exosomes. Here we investigated if these lipid vesicles were indeed exosomes that have an endosomal biogenesis.

**Method:**

In most cells, endocytosis is thought to occur at specialized microdomains known as lipid rafts. To demonstrate an endosomal origin for MSC exosomes, MSCs were pulsed with ligands e.g. transferrin (Tfs) and Cholera Toxin B (CTB) that bind receptors in lipid rafts. The endocytosed ligands were then chased to determine if they were incorporated into the exosomes.

**Results:**

A fraction of exogenous Tfs was found to recycle into MSC exosomes. When MSCs were pulsed with labelled Tfs in the presence of chlorpromazine, an inhibitor of clathrin-mediated endocytosis, Tf incorporation in CD81-immunoprecipitate was reduced during the chase. CTB which binds GM1 gangliosides that are enriched in lipid rafts extracted exosome-associated proteins, CD81, CD9, Alix and Tsg101 from MSC-conditioned medium. Exogenous CTBs were pulse-chased into secreted vesicles. Extraction of Tf- or CTB-binding vesicles in an exosome preparation mutually depleted each other. Inhibition of sphingomyelinases reduced CTB-binding vesicles.

**Conclusion:**

Together, our data demonstrated that MSC exosomes are derived from endocytosed lipid rafts and that their protein cargo includes exosome-associated proteins CD81, CD9, Alix and Tsg101.

## Introduction

Mesenchymal stem cells (MSCs) are multipotent fibroblast-like cells that are readily prepared from both adult and foetal tissues (1–6), human embryonic stem cells (ESCs) (7, 8) and induced pluripotent stem cells (iPSCs) (9). They are currently the stem cell of choice for regenerative medicine and are being evaluated in at least 306 trials (http://www.clinicaltrials.gov/; accessed March 2013) for a myriad of diseases that include many cardiovascular diseases, osteogenesis imperfecta (OI), amyotrophic lateral sclerosis (ALS), lysosomal storage diseases, steroid refractory Graft versus Host Disease (GvHD) and bone fractures (10). The popularity of MSCs could be attributed to their easy availability from ethically and socially acceptable tissues, their large *ex vivo* expansion capacity (10) and a reportedly large differentiation potential to generate not only mesodermal cell types such as adipocytes, osteocytes and chondrocytes (11–16) but also endothelial cardiovascular and neurogenic cell types (17–23). However, the therapeutic efficacy of MSCs has been increasingly attributed to their secretion, which is thought to reduce cellular injury and enhance repair (24).

Consistent with this secretion hypothesis for MSC therapeutic efficacy, our group observed several years ago that culture medium conditioned by human embryonic stem cell-derived MSCs (hESC-MSCs) when administered intravenously prior to reperfusion reduced infarct size in a pig and mouse model of ischemia/reperfusion injury (25). Size fractionation of the conditioned medium subsequently revealed that the active component had a presumptive size of 50–200 nm and this component was then isolated by size exclusion high performance liquid chromatography (HPLC) (26). At that time, we described these particles as exosomes on the basis that they are homogenously sized particles with a hydrodynamic radius of 55–65 nm, a flotation density in sucrose of 1.10–1.18 g/mL, a cargo of exosome-associated proteins such as the tetraspanin proteins, CD9 and CD81, Alix and Tsg101. They also contained RNAs that were primarily short RNAs of less than 300 nt with many microRNAs that were mostly pre-microRNAs (27). These purified particles reduced infarct size to the same extent as chemically defined medium (CM) but at one-tenth of the protein dosage used in CM.

To better understand the physiological importance and function of these secreted MSC vesicles, we investigate their biogenesis to determine if these vesicles were indeed exosomes that is, extracellular vesicles that have an endosomal origin. Exosome, unlike the other extracellular vesicles described to date, is the only secreted vesicle to have an endosomal biogenesis. During exosome biogenesis, the membrane of endosomes invaginates to form numerous intraluminal vesicles (ILVs) within a membrane vesicle, resulting in a multivesicular body (MVB). When MVB and plasma membrane fuse, the ILVs are released into the extracellular space as exosomes (28). Consequently, exosomes are characterized by a conserved set of proteins that are associated with endocytosis and endosomal trafficking such as caveolins, clathrin, transferrin receptors (TfRs), tetraspanins (CD81, CD63, CD9), Alix and Tsg101, and so on (29).

To determine if MSC has an endosomal origin, we tracked labelled extracellular ligands such as transferrins (Tfs) and cholera-toxin B chains (CTBs) that are classical ligands for endocytosis to examine newly produced exosomes for the presence of these labelled ligands. Tfs are generally internalized by TfRs in the classical receptor-mediated endocytosis to form endosomes. After the 2 ferric ions bound in Tf are released, the apotransferrin which remained bound to the receptor in the endosome is recycled to the cell membrane. As some endosomes are shuttled into exosome biogenesis, some of the receptor-bound Tfs in the membrane of endosomes would be incorporated into the membrane of exosomes. CTBs, on the other hand, are ligands for GM1 gangliosides (30) which are present predominantly in lipid rafts (31). Lipid rafts are structurally and functionally unique microdomains in the plasma membrane and are generally sites for important membrane activities such as endocytosis, cell signalling, cell adhesion and membrane trafficking [reviewed (32)] and endocytosis (33). Like Tfs, exogenous CTBs would be expected to be incorporated in exosomes.

Lipid raft is also enriched in cholesterol and saturated phospholipids such as sphingolipids. In particular, sphingolipid ceramide has been shown to be important in the sorting of exosome-associated plasma membrane domains into the lumen of the endosome during exosome biogenesis (34).

In this study, we specifically investigated if biogenesis of our MSC exosome is associated with endocytic activity by tracking endocytosed labelled Tf or CTB, and if exosome production was dependent on sphingolipid by treating MSC with a sphingomyelinase inhibitor.

## Materials and methods

### MSC culture

Immortalized E1-*MYC* 16.3 human ESC-derived MSCs were cultured in DMEM with 10% foetal calf serum as previously described (35). For exosome preparation, the cells were grown in a CM for 3 days and exosomes were purified from conditioned medium by size exclusion on HPLC as previously described (26, 36, 37). The CM was harvested, concentrated 50× by tangential flow filtration (TFF) using a membrane with a 100 kDa MWCO (Sartorius, Goettingen, Germany) and then fractionated by HPLC (TSK Guard column SWXL, 6×40 mm and TSK gel G4000 SWXL, 7.8×300 mm, Tosoh Corp., Tokyo, Japan). The first eluted peak was collected and concentrated using 100 kDa MWCO filter (Sartorius, Goettingen, Germany). The purified exosomes were filtered with a 0.22 µm filter (Millipore, Billerica, MA) and stored at −20°C in a freezer until use.

### Tf uptake

To visualize Tf uptake, 4-chamber slides were coated with 0.1% (w/v) gelatin. Approximately 5,000 E1-*MYC* 16.3 MSCs were plated into each chamber and incubated overnight. The cells were washed twice with PBS and incubated with 500-µL serum-free medium for 1 hour. Tf-A488 (Life Technology, Grand Island, NY) was added into each well to a final concentration of 1, 5, 10 or 50 µg/mL. After 15 minutes, the cells were fixed with 500 µL of 4% paraformaldehyde per well for 10 minutes and counterstained with 4’, 6-diamidino-2-phenylindole (DAPI)for 10 minutes. After washing twice with PBS, the cells were visualized by confocal microscopy. To assess uptake and release of biotinylated Tf, Tf-Biotin (Life Technology, Grand Island, NY) was added to the chemically defined culture medium (37) to a final concentration of 5 µg/mL prior to conditioning. The medium was harvested after 24, 48 and 72 hours of conditioning and purified as described above.

### Cellular uptake of Tf and CTB

To determine the uptake of Tf and CTB by MSCs, 10^5^ E1-*MYC* 16.3 MSCs were plated onto each well of a 6-well culture plate and incubated overnight. The cells were then treated with 5 µg/mL of either Tf-A488 (Life Technology, Grand Island, NY) or CTB-A488 and incubated for 15, 30, 60, 120 and 240 minutes before flow cytometry analysis.

### CPZ inhibition of endocytosis

To assess if clathrin-mediated endocytosis was involved in the internalization of Tf by E1-*MYC* 16.3, MSCs were plated into each well of a 6-well culture plate and incubated overnight. The cells in each well were starved in 2 mL of serum-free medium for 1 hour before exposure to 0, 7.5, 15 or 30 µM chlorpromazine (CPZ) for 30 minutes. The cells were washed with PBS and incubated for another 30 minutes with fresh serum-free medium containing 5 µg/mL of Tf-A488 and 0, 7.5, 15 or 30 µM CPZ. The Tf-A488-treated cells were harvested and analysed by flow cytometry on a BD FACSCalibur flow cytometer. In another experiment, 10^5^ E1-*MYC* 16.3 cells were plated into each well of a 6-well culture plate. After overnight incubation, the cells were serum-starved in CM (37) for 1 hour before exposure to 0, 7.5, 15 or 30 µM CPZ for 30 minutes, then washed and incubated in fresh medium containing 5 µg/mL of Tf-Biotin and 0, 7.5, 15 or 30 µM CPZ for another 30 minutes. The cells were washed again and incubated in CM (37) for another 6 hours. The conditioned medium was harvested, concentrated, immunoprecipitated with antiCD81 antibody and probed for the presence of CD9 and Tf-Biotin by western blot.

### CTB-binding assay

CTB (SBL Vaccin AB, Sweden) was biotinylated using Sulfo-NHS Biotin (Thermo Scientific, Rockford, IL) as per manufacturer's instruction. CM (20 µg) from E1-*MYC* 16.3 MSCs were incubated with 0.1 µg biotinylated CTB in 100 µL PBS pH7.4 for 30 minutes with rotation. A 5 µL of Dynabeads M280 streptavidin (Life Technology, Grand Island, NY) that were washed as described above was added to the exosome–CTB reaction mix and incubated by shaking at 800 rpm for 30 minutes. The beads were immobilized with a magnet, and the supernatant was removed and labelled as the “unbound” fraction. The beads were then washed twice with 100 µL Wash Buffer (0.1% BSA in PBS) as described above and the supernatants were labelled as “wash1” and “wash2,” respectively. The beads were resuspended in 100 µL PBS to represent the bound fraction. Four micrograms of CM and 20 µL each of the “unbound,” “wash1,” “wash2” and “bound” fractions were denatured and resolved on a 4–12% SDS-polyacrylamide gel before analysis using western blot hybridization.

## CD81 immunoprecipitation

Dynabeads M-280 sheep anti-mouse IgG (100 µL) (Life Technology, Grand Island, NY) were washed as described above and then incubated with 10 µL mouse anti-human CD81 antibody (Santa Cruz, CA) for 2 hours with gentle shaking at room temperature. The antibody-bound dynabeads were washed twice and incubated with 20 µg CM for 2 hours with rotation at room temperature. The CD81 immunoprecipitate was washed twice, denatured and resolved on a 4–12% SDS-polyacrylamide gel before analysis using western blot hybridization.

### Western blot hybridization

Western blot hybridization was performed using standard protocols. Briefly, proteins were denatured, separated on 4–12% polyacrylamide gels, electroblotted onto a nitrocellulose membrane and probed with a primary antibody followed by horseradish peroxidase-coupled secondary antibodies against the primary antibody. The primary antibodies used in this study were mouse anti-human CD71, CD9, ALIX and TSG101 (Santa Cruz, CA). For detection of biotinylated Tf, the blot was probed using a streptavidin-horseradish peroxidase conjugate. The blot was then incubated with a chemiluminescent HRP substrate to detect bound primary antibody, and therefore the presence of the antigen.

### CTB or Tf coupled CD81 ELISA assay

MSC CM (10 µg) was incubated with either 0.5 ηg biotinylated CTB or 2 µg biotinylated Tf (Life Technology, Grand Island, NY) in a final volume of 100 µL. Ligand-bound vesicles were extracted using streptavidin-conjugated magnetic beads as described earlier. The bound vesicles were then incubated with 100 µL of 1: 500 diluted anti-CD81 antibodies (Santa Cruz, CA) and 1:5000 diluted HRP-conjugated goat anti-mouse secondary antibodies. HRP activity was determined using Amplex Red Substrate (Life Technology, Grand Island, NY) as per the manufacturer's protocol.

### Sphingomyelinases (nSMases) inhibition

To determine if ceramide is important in MSC exosome biogenesis, 10^5^ E1-MYC 16.3 cells were plated into each well of a 6-well culture plate. After overnight incubation, the cells were incubated with serum-free medium containing 0, 2.5, 5 or 10 µM GW4869 (nSMases Inhibitor) overnight. Following treatment, medium was replaced with serum-free defined medium and cells were incubated overnight. The CM was harvested and concentrated using a 30-kDa MWCO filter (Merck Millipore). Ten micrograms of the concentrated medium was resolved on a protein gel before probing for ALIX, TSG101 and CD9 by western blot. Ten micrograms of the concentrated medium was analysed using CTB-coupled CD81 ELISA assay as described earlier.

### Sucrose gradient

A 22.8–60% sucrose density gradient was prepared as previously described (26). MSC CM (250 µg) was loaded on the top of the gradient before ultracentrifugation for 16.5 hours at 200,000×*g*, 4°C in a SW60Ti rotor (Beckman Coulter Inc., CA). After centrifugation, 13 fractions were collected starting from the top of the gradient. The densities of each were determined by weighing a fixed volume. Protein concentration of each fraction was quantified using NanoOrange Protein Quantification kit, (Life Technology, Grand Island, NY) according to the manufacturer's instructions. Each fraction was then assayed for the presence of CD81 and CTB-binding activity using a CTB-CD81 coupled ELISA. A total of 50 µL of each fraction was incubated with 0.5 ηg biotinylated CTB in 100 µL PBS pH 7.4 to extract CTB-bound vesicles as described above. The bound vesicles on magnetic beads were then incubated with 100 µL 1: 500 diluted HRP-conjugated anti-CD81 antibodies (Santa Cruz, CA). Detection was done using 100 µL Amplex Red Substrate (Life Technology, Grand Island, NY) and fluorescent intensity was measured at 530/590 nm (Ex/Em).

## Pulse chase of CTB

E1-MYC 16.3 cells (3×10^6^) were plated into a 10-cm tissue culture dish and incubated overnight. The cells were washed and incubated in medium containing 5 µg/mL biotinylated CTB for 30 minutes. At 0.5, 1, 2, 4, 8, 16 and 32 hours, the medium was harvested and replaced with fresh CM. The harvested CM was concentrated with a 30-kDa MWCO filter and assayed using CTB-coupled CD81 ELISA.

## Electron microscopy

A drop of 1 ng/mL of exosome in PBS was placed on a formvar-carbon-coated nickel grid for 1 hour and then washed with 0.1 M sodium cacodylate, pH 7.6 before fixing in 2% paraformaldehyde and 2.5% glutaraldehyde in 0.1 M sodium cacodylate, pH 7.4 for 10 minutes. After washing with 0.1 M sodium cacodylate, pH 7.6, contrast sample with 2% uranyl acetate in 0.1 M sodium cacodylate, pH 7.4 for 15 minutes, washed and incubated with a drop of 0.13% methyl cellulose and 0.4% uranyl acetate for 10 minutes. After air-drying for 5 minutes, the sample was examined with JEM-2200FS electron microscope operated at 100 kV.

## Results

### Extracellular Tf was internalized by MSCs through clathrin-mediated endocytosis and was partially recycled in exosomes

**Fig. 1:**
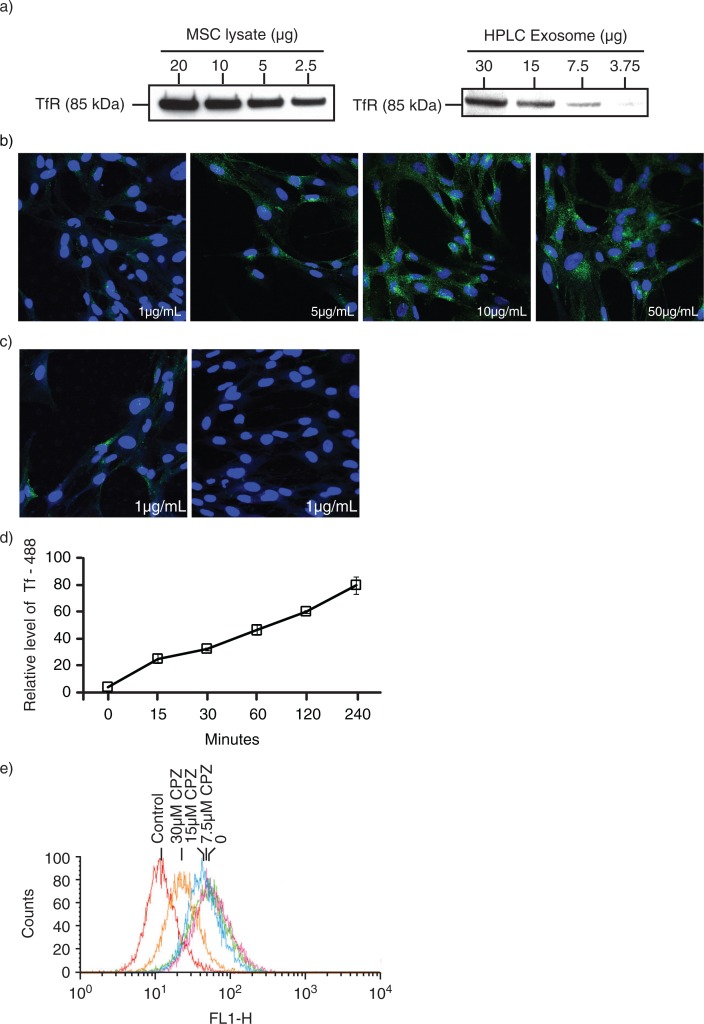
Transferrin (Tf) uptake and release by mesenchymal stem cells. a) Western blot analysis for the presence of CD71 (TfRs) in E1-*MYC* 16.3 cell lysate and HPLC-purified exosome preparations using anti-CD71; b) E1-*MYC* 16.3 cells were cultured in chamber slides. After incubating with 1, 5, 10 or 50 µg/mL of Alexa488-labelled Tf for 15 minutes, the cells were washed, fixed, counterstained with DAPI and observed by confocal microscopy. c) E1-*MYC* 16.3 cells were cultured in the presence of 1 µg/mL of Alexa488-labelled Tf for 30 minutes. The cultures were washed. Half of the cultures were fixed and counterstained with DAPI. The other half were cultured for another 30 minutes in the absence of the labelled Tf before they were similarly processed and observed by confocal microscopy; d) 10^5^ E1-*MYC* 16.3 cells were plated into each well of a 6-well culture plate. After overnight incubation, cells were treated with 5 µg/mL of Tf-A488 (Life Technology, Grand Island, NY) and incubated for 15, 30, 60, 120 and 240 minutes. The cells were harvested and analysed by flow cytometry. e) 10^5^ E1-*MYC* 16.3 cells were plated into each well of a 6-well culture plate. After overnight incubation, the cells in each well were serum-starved for 1 hour, exposed to 0, 7.5, 15 or 30 µm CPZ before washing with PBS and incubated for another 30 minutes with fresh serum-free medium containing 5 µg/mL of Tf-A488 and 0, 7.5, 15 or 30 µm CPZ. The cells were harvested and analysed by flow cytometry.

To test if MSC exosomes were derived from endosomes, we first confirmed the presence of TfRs in MSCs and HPLC-purified MSC exosome preparations (Fig. 1a). TfRs are often used as the representative receptor for clathrin-mediated endocytosis and their presence in MSC exosomes had been previously reported to be present by mass spectrometric analysis of MSC exosome proteome (38, 39). When MSCs were incubated with different concentrations of labelled ligands for TfRs, that is, fluorescent Alexa 488–labelled Tf, most cells were labelled by 15 minutes (Fig. 1b). Within 30 minutes of removing the labelled Tf from the culture medium, the fluorescence in the cells was lost (Fig. 1c). This Tf uptake was time dependent (Fig. 1d). Incubation with CPZ, an inhibitor of clathrin-mediated endocytosis inhibited cellular uptake of fluorescent Tf in a concentration-dependent manner (Fig. 1e). Therefore, MSC internalized Tf through clathrin-mediated endocytosis, and the internalized Tf was turned over, possibly recycled to the extracellular milieu within 30 minutes.

**Fig. 2:**
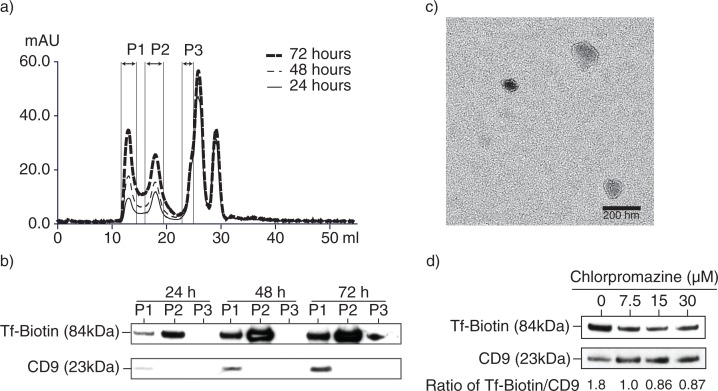
Isolation of exosomes from transferrin (Tf)-fed MSCs. a) To assess uptake and release of biotinylated Tf, E1-*MYC* 16.3 cells were grown in chemically defined serum-free culture medium containing biotinylated Tf. The medium was harvested after 24, 48 and 72 hours of conditioning and purified by HPLC as described in Materials and Methods. The HPLC chromatogram illustrated the resolution of the components into 3 peaks in each of the conditioned medium. b) Western blot analysis of the 3 peaks in each conditioned medium for the presence of biotinylated Tf and CD9 using streptavidin-HRP and anti-CD9 antibody, respectively. Equal volume of each peak was loaded in each lane. c) TEM images of HPLC Peak 1, scale bar=200 ηm. d) 10^5^ E1-*MYC* 16.3 cells were plated into each well of a 6-well culture plate. After overnight incubation, the cells were starved in chemically defined serum-free culture medium for 1 hour before exposure to 0, 7.5, 15 or 30 µM chlorpromazine (CPZ) for 30 minutes. The cells were washed with PBS and incubated for another 30 minutes with fresh serum-free medium containing 5 µg/mL of Tf-Biotin and 0, 7.5, 15 or 30 µM CPZ. The medium was then removed and replaced with fresh defined medium. After conditioning for 6 hours, the medium was collected and concentrated using an Amicon 100 kDa filter. The concentrated medium was immunoprecipitated with anti-CD81 antibody. The immunoprecipitate was analysed by western blot hybridization and probed for biotinylated Tf using streptavidin-conjugated HRP and CD9 using an anti-CD9 antibody. The signal ratio of the biotinylated Tf to CD9 is indicated for each concentration of CPZ.

Next, we determined if part of the endocytosed Tfs were recycled in exosomes. MSCs were incubated with biotinylated Tf and aliquots of the culture medium were removed at 24, 48 and 72 hours. Exosomes were purified from the CM by HPLC size exclusion fractionation as previously described (26). Peak 1, which represents cumulative exosome fraction, increased as expected with the duration of conditioning (Fig. 2a). This exosome-enriched peak was confirmed by the presence of CD9 (Fig. 2b) and the presence of bilipid membrane vesicles as observed by electron microscopy (Fig. 2c). Biotinylated Tfs are present in peak 1 and 2 with increasing quantity over time. We hypothesized that most of the biotinylated Tf was internalized within the first 24 hours and then released over time by the cells, resulting in the accumulating concentration of biotinylated Tf. As evidenced by the relatively large amount of biotinylated Tf in Peak 2 (Fig. 2b), most of the Tf was released in the soluble form and only a small fraction was found in Peak 1 or exosomes.

To confirm that at least some of the Tfs in the exosome fraction were indeed endocytosed and then incorporated into exosomes during exosome biogenesis, and not bound by the receptor on newly secreted exosomes, MSCs were incubated with biotinylated Tfs in the presence or absence of CPZ. The biotinylated Tfs and CPZ were then removed and the cells were incubated with fresh medium for another 6 hours. The conditioned medium was immunoprecipitated with an anti-CD81 antibody. CPZ reduced the presence of biotinylated Tfs relative to CD9 in the anti-CD81 immunoprecipitate (Fig. 2d). This suggests that incorporation of Tf into MSC exosomes is dependent on clathrin-mediated endocytosis.

### MSC exosomes possess characteristic features of lipid raft, a microdomain with active endocytic activity in the plasma membrane

**Fig. 3:**
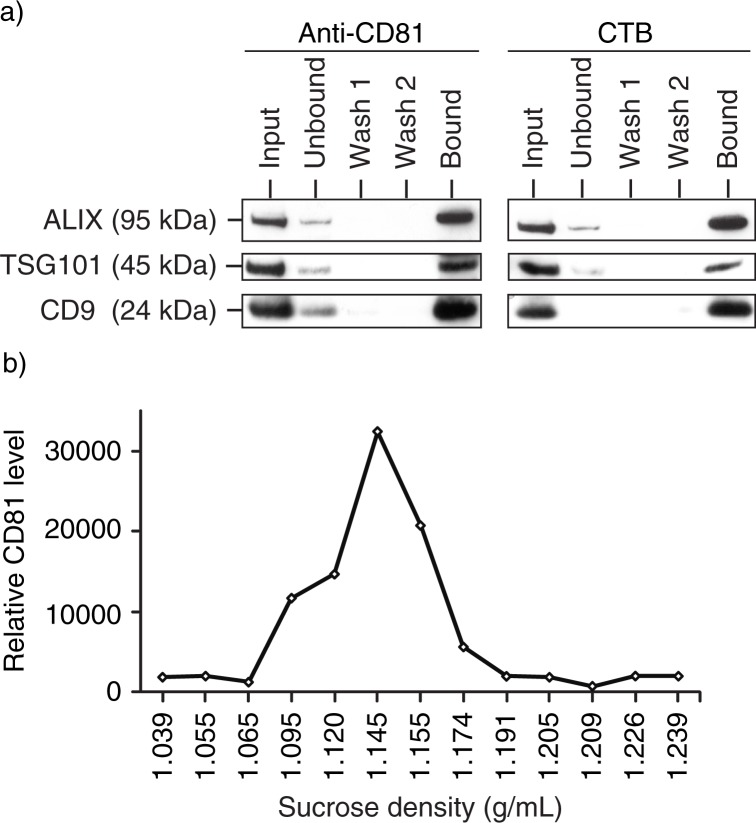
Presence of CTB-binding domains in MSC exosomes. a) MSC exosomes were incubated with either biotinylated CTB or biotinylated CD81 antibody and extracted using streptavidin-conjugated beads. The beads were boiled in SDS-PAGE loading buffer and the proteins resolved by SDS-PAGE in a 4–12% gradient gel. After electroblotting the resolved proteins onto a nitrocellulose membrane, the proteins were probed for ALIX, TSG101 and CD9. b) 250 µg MSC CM was loaded on the top of a 22.8–60% sucrose density gradient and ultracentrifuged for 16.5 hours at 200,000×*g*, 4°C. After centrifugation, 13 fractions were collected starting from the top of the gradient. The density of each fraction was determined by weighing a fixed volume. Fifty microlitres of each fraction was then incubated with 0.5 ηg biotinylated CTB in 100 µL PBS pH7.4 to extract CTB-bound vesicles using streptavidin-conjugated magnetic beads. The bound vesicles on magnetic beads were then incubated with 100 µL 1: 500 diluted HRP-conjugated anti-CD81 antibodies to assay for the relative level of CD81. The level of CD81 was normalized to that in the lightest fraction, that is, 1.039 g/mL.

As endocytic activity in cells is concentrated at specialized microdomains in the plasma membranes such as lipid rafts which enriched in GM1 gangliosides, we determined here if MSC exosomes have GM1 gangliosides. Since GM1 gangliosides are the cellular receptors for CTB (40), we determine the presence of lipid rafts in MSC exosomes by assessing the affinity of MSC exosomes for CTB. MSC exosomes were incubated with biotinylated CTB and the CTBs were then extracted using streptavidin-conjugated magnetic beads. The beads were resolved by SDS-PAGE and analysed by western blot analysis for exosome markers, ALIX, TSG101 and CD9 (Fig. 3a). A parallel immunoprecipitation of the same exosome preparation by anti-CD81, an antibody against another exosome-associated tetraspanin protein, also precipitated ALIX, TSG101 and CD9, demonstrating that these proteins, namely CD81, ALIX, TSG101 and CD9, resided on the same complex as GM1 gangliosides. As GM1 ganglioside is a phospholipid and the proteins were either membrane or cytosolic proteins, the complex is likely to be a phospholipid membrane vesicle.

To determine if CTB-bound vesicles have the same flotation density in sucrose as MSC exosomes, conditioned medium was fractionated on a sucrose gradient and each fraction was extracted with CTB (Fig. 3b). The CTB extracts were then assayed for CD81. The CTB-bound CD81 had a flotation density of 1.09–1.17 g/mL. Together, these observations demonstrated the enrichment of GM1 gangliosides in the membrane of MSC exosomes, and this enrichment is consistent with exosome biogenesis from endosomes which are generally derived from lipid rafts.

### GM1 gangliosides are integral components of MSC exosomes

**Fig. 4:**
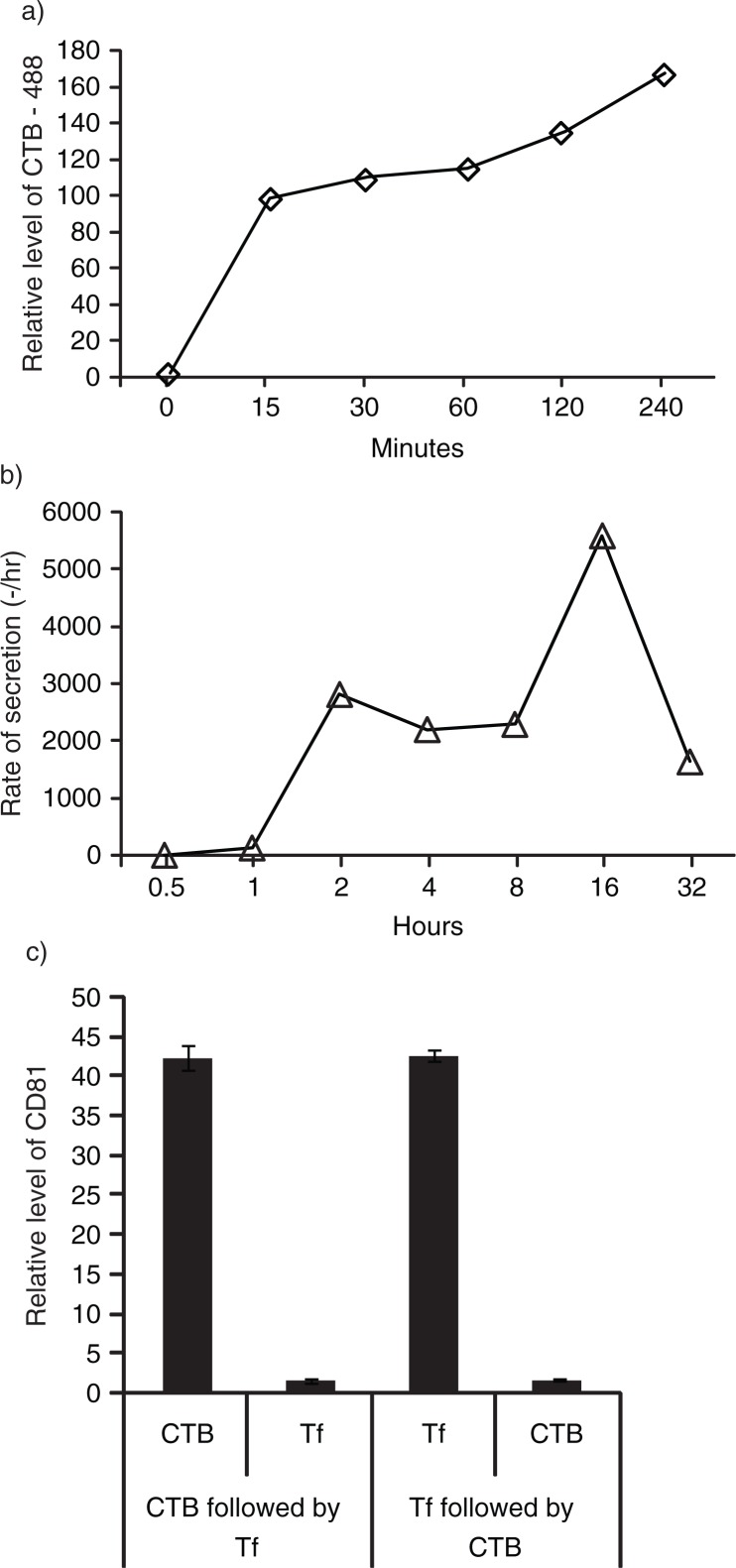
GM1 gangliosides and TfRs are integral components of exosomes. a) After overnight incubation of 10^5^ E1-*MYC* 16.3 cells in each well of a 6-well culture plate, the cells were treated with 5 µg/mL of CTB-488 and incubated for 15, 30, 60, 120 and 240 minutes before flow cytometry analysis; b) after overnight incubation of 3×10^6^ E1-MYC 16.3 cells in a 10-cm tissue culture dish, the cells were washed and incubated in medium containing 5 µg/mL biotinylated CTB for 30 minutes before washing and replacing culture with fresh medium. At 0.5, 1, 2, 4, 8, 16 and 32 hours, the medium was harvested and replaced with fresh medium. The harvested CM was concentrated and assayed for CTB-coupled CD81 by ELSA; c) To confirm the co-localization of GM1 gangliosides and TfRs in exosomes, HPLC-exosome fraction was extracted with excess CTB or Tf. Relative levels of CTB- and Tf-binding vesicles were determined by measuring CD81. The extracted fractions were further extracted with Tf or CTB, respectively. These second extracts were also assayed for CD81.

CTB is known to have a high specificity for GM1 gangliosides (30) which are enriched in lipid rafts. To confirm that the membrane of MSC exosomes was derived from endocytosed lipid rafts, fluorescent CTB was used to label the lipid rafts on MSC. MSCs internalized the labelled CTB in a time-dependent manner (Fig. 4a). To track if internalized CTBs are incorporated in exosomes, MSCs were pulsed with 5 µg/mL biotinylated CTB for 30 minutes and washed, and the medium was replaced with fresh medium. The labelled CTB was then chased for 32 hours during which the culture medium was harvested and replaced with fresh medium at different time points. The biotinylated CTB-bound CD81 level in harvested medium was assayed by ELISA and normalized to the time of incubation prior to harvest. The rate of biotinylated CTB-bound CD81 secretion in the medium increased over time to peak at 16 hours, suggesting that MSC exosomes were derived from endocytosed lipid raft (Fig. 4b).

### GM1 gangliosides and TfRs co-localized in secreted vesicles

Since TfRs and GM1 gangliosides are both localized in lipid rafts, we hypothesized that both also co-localized on the same exosomes. To confirm the co-localization of GM1 gangliosides and TfRs in exosomes, HPLC-exosome fraction was extracted with excess CTB or Tf to remove any vesicles containing GM1 gangliosides and TfRs, respectively. Relative levels of CTB- and Tf-binding vesicles as determined by measuring CD81 were similar (Fig. 4c). After extraction with CTB or Tf, the extracted fractions were further extracted with Tf or CTB, respectively. The extracts were then assayed for CD81. The first extraction with excess CTB or Tf extracted similar amounts of CD81and effectively depleted CD81 such that the remaining CD81 constituted only 4% of the total (Fig. 4c). This demonstrated that the CTB- and Tf-binding vesicles are the same vesicles.

### Production of MSC exosomes is dependent on sphingomyelinase

**Fig. 5:**
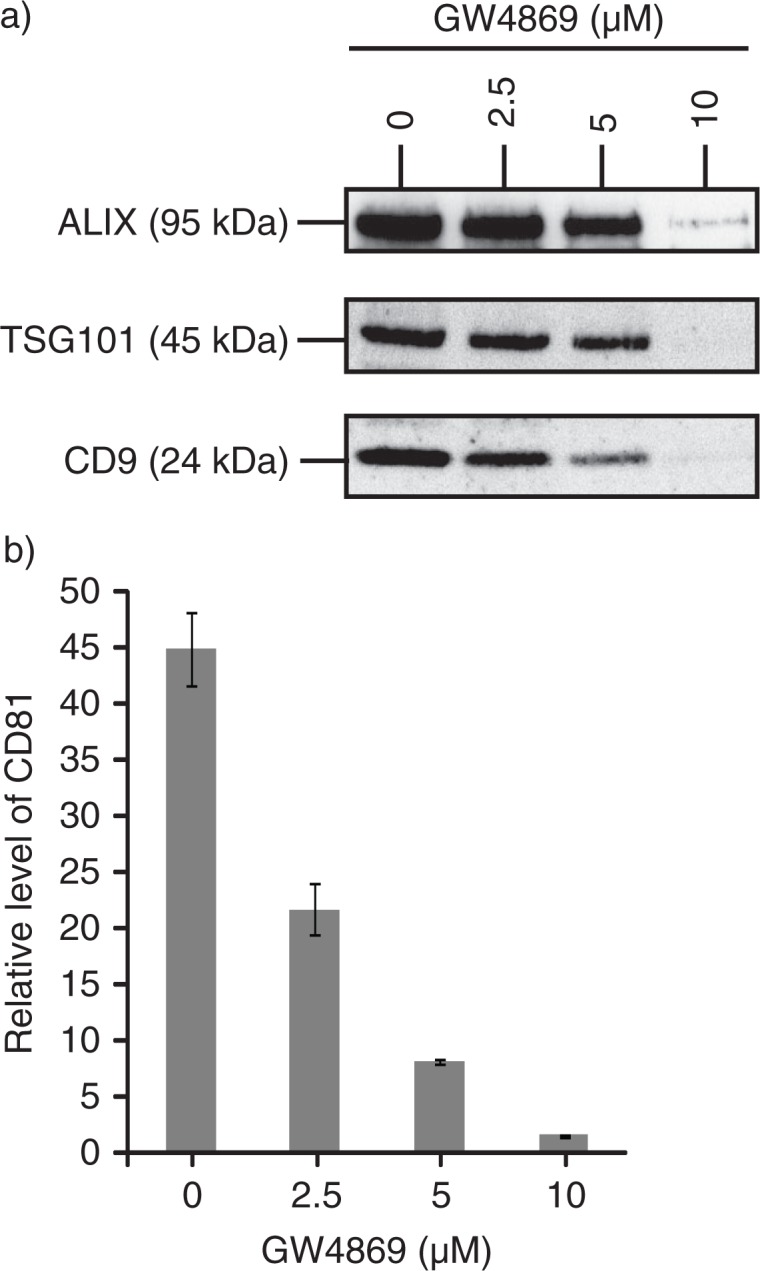
MSC exosome production is dependent on sphingomyelinase. 10^5^ E1-MYC 16.3 cells were plated into each well of a 6-well culture plate, treated with 0, 2.5, 5 or 10 µM GW4869 (nSMases Inhibitor) for 1 hour, washed and incubated overnight. The conditioned medium was harvested and concentrated. The concentrated medium was analysed by a) western blot (25 µg); and b) CTB-coupled CD81 ELISA assay (10 µg).

Sphingomyelinase which hydrolysed sphingomyelin to ceramide had been reported to control the exosome biogenesis and secretion independent of the ESCRT pathway (41). To evaluate the importance of sphingomyelinase in the biogenesis of MSC exosomes, MSCs were treated with GW4869, an inhibitor of nSMases. The secretion of ALIX, TSG101 and CD9 into the culture medium was reduced with increasing concentration of GW4869 (Fig. 5a). GM1 + vesicles as measured by the level of CD81 in CTB-binding vesicles in the CM were also decreased (Fig. 5b). This demonstrated that the production of MSC exosomes is dependent on sphingomyelinase and this is consistent with the hypothesis that budding of exosomes in MVB endosomes requires ceramide (41).

## Discussion

In this report, we investigated if our previously described MSC exosome, a HPLC-purified fraction, had an endosome-dependent biogenesis. Specifically, we first investigated if exogenous ligands such as Tfs and CTBs are incorporated in exosomes after endocytosis. Tfs are generally internalized via TfRs, the archetypical receptor for clathrin-mediated endocytosis. When MSCs were incubated with labelled Tfs, they produced exosomes with labelled Tfs. We also observed that pulsing MSCs with labelled Tfs in the presence of CPZ reduced the incorporation of Tfs in anti-CD81-immunoprecipitate. On the other hand, CTB bind GM1 gangliosides that are enriched in lipid rafts. As lipid rafts are microdomains in plasma membrane that are also sites of active cellular activities, including clathrin-mediated endocytosis, the presence of lipid raft components in MSC exosomes would further support the endosomal origin of MSC exosomes. Here we demonstrate that CTB could extract exosome-associated proteins from MSC-conditioned medium and HPLC-purified exosome preparation, and this CTB extract was found in a fraction that had the same flotation density of exosome in sucrose. When pulse-labelled with biotinylated CTBs, MSCs produced biotinylated exosomes during a 16-hour chase. Production of CTB-binding exosomes was also found to be sensitive to GW4869, an inhibitor of sphingomyelinase. Finally, CTB-binding activity was found to co-localize with TfRs, such as extraction of MSC exosome preparation with CTB or Tf depletes Tf- or CTB-binding activity, respectively.

Together, this study demonstrated that MSC exosomes are derived from the endocytosis at the lipid rafts of plasma membrane.
